# Long-Term Prognosis of Patients With Anti-N-Methyl-D-Aspartate Receptor Encephalitis Who Underwent Teratoma Removal: An Observational Study

**DOI:** 10.3389/fneur.2022.874867

**Published:** 2022-04-12

**Authors:** Hesheng Zhang, Weixi Xiong, Xu Liu, Wenyu Liu, Dong Zhou, Xintong Wu

**Affiliations:** Department of Neurology, West China Hospital of Sichuan University, Chengdu, China

**Keywords:** anti-NMDAR encephalitis, teratoma, surgery, prognosis, relapse

## Abstract

**Objective:**

This study aimed to evaluate the clinical characteristics and long-term surgical outcomes of patients with anti-N-methyl-D-aspartate receptor (NMDAR) encephalitis with teratoma.

**Methods:**

Patients who were admitted to West China Hospital from June 2012 to June 2019 and diagnosed with anti-NMDAR encephalitis were enrolled in the study. Medical records were reviewed prospectively to gather clinical characteristic data. Patients were followed up at long-term every 3 months.

**Results:**

This study included 192 patients, among whom 21 (10.9%) were detected with having a teratoma. Patients included 20 women, with a mean age of 24.62 ± 7.61 years. Seizure and psychiatric symptoms were the most dominant symptoms in both groups, followed by memory deficits. Central hypoventilation (52.4 vs. 17%, *p* < 0.001) and decreased consciousness (71.4 vs. 31.3%, *p* = 0.002) were significantly more frequent in patients with teratoma than in those without. Moreover, the anti-NMDAR antibody titer was higher (*p* = 0.021) and the baseline modified Rankin scale score was lower (*p* = 0.004) in patients with teratoma than in those without. First-line immunotherapy was performed in 21 (100%) patients with teratoma and 167 (97.7%) patients without teratoma. All patients with teratoma had the tumor removed. During follow-up, two (9.5%) patients with teratoma and 11 (6.4%) patients without teratoma died, whereas 1 (4.8%) patient with teratoma and 37 (21.6%) patients without teratoma had relapses. Overall, 19 (90.5%) patients with teratoma and 151 (88.3%) patients without teratoma achieved favorable clinical outcomes at the final follow-up.

**Conclusions:**

With early detection and removal of teratoma, most patients with anti-NMDAR encephalitis and teratoma achieved a favorable long-term prognosis.

## Introduction

Anti-N-methyl-D-aspartate receptor (NMDAR) encephalitis is an autoimmune disease that is characterized by psychiatric symptoms, seizures, memory deficits, speech impairment, movement disorders, autonomic instability, central hypoventilation, and decreased consciousness (1–3). The condition is primarily mediated by specific immunoglobulin G (IgG) antibodies against the NR1 subunit of the NMDAR. Tumors (mainly teratomas) containing nerve tissues can induce the production of specific antibodies *via* molecular mimicry and have been identified as a trigger of anti-NMDAR encephalitis (3, 4). Previous studies have reported a prevalence of teratoma at 20.2–45% in patients with anti-NMDAR encephalitis (5, 6). Immunotherapy is the most crucial therapeutic method for anti-NMDAR encephalitis. For patients with a tumor, surgery is an important treatment strategy and is recommended to be performed as soon as possible (3). In this study, we explored the clinical characteristics and long-term prognoses of these patients, following a surgery.

## Methods

### Study Design and Participants

The Outcome of anti-NMDAR Encephalitis Study in Western China (ONE-WC) study was registered with the WHO international clinical trial registry platform (registration number: ChiCTR1800019762) and is described in more detail in our previous publications (7–11). Patients were hospitalized patients recruited from the Neurology Department of West China Hospital from October 2011 to June 2019. Inclusion criteria were as follows: (1) rapid onset of at least one of eight major groups of symptoms (psychosis, memory deficits, speech disturbances, seizures, movement disorders, disturbance of consciousness, autonomic dysfunctions, and central hypoventilation) (1); (2) positive for anti-NMDAR antibodies in the cerebrospinal fluid (CSF).

Exclusion criteria were as follows: (1) human immunodeficiency virus infection, meningitis, brain abscess, prior diseases, cerebral malaria, brain tumor, or diagnosis of a non-infectious central nervous system disease, such as acute demyelinating encephalomyelitis; (2) patients with laboratory evidence of infectious encephalitis; (3) patients diagnosed with epilepsy, cerebral trauma, and/or other nervous system diseases prior to the onset of encephalitis; (4) patients with other coexisting positive autoimmune or neurologic paraneoplastic antibodies, such as α-amino-3-hydroxy-5-methyl-4-isoxazolepropionic acid receptors-1 and−2, contactin-associated protein-2, leucine-rich glioma-inactivated protein-1, c-aminobutyric acid receptor B1/B2, anti-neuronal nuclear antibody (ANNA)-1, ANNA-2, and Purkinje-cell cytoplasmic autoantibody-1.

### Clinical Management

A lumbar puncture was performed in suspected patients with rapid onset of neurological or psychiatric disorders. Samples were assessed using an indirect immunofluorescence assay for the detection of autoimmune neurologic paraneoplastic antibodies. Individuals with confirmed antibodies underwent chest and abdomen CT or abdomen and reproductive system ultrasound to search for potential tumors. Abnormalities were reported by radiologists and reviewed by relevant specialists (e.g., gynecologists for a pelvic mass in women).

Treatments were administered by senior neurologists of West China Hospital, Department of Neurology. Immunotherapies included first-line immunotherapy (intravenous immunoglobulin [IVIg], methylprednisolone, and plasma exchange) and second-line immunotherapies (rituximab, cyclophosphamide, azathioprine, mycophenolate mofetil, and tacrolimus). First-line immunotherapies were administered as follows: .4 g/kg IVIg was administered daily for 5 days as one turn; 1,000 mg intravenous methylprednisolone was administered daily for 3–5 days as one turn, then replaced by daily prednisone. Repeated intravenous immunotherapy was administered to patients with a poor response. Other interventions included anti-epileptic drugs, anti-psychotic drugs, sedative-hypnotic drugs, and other symptomatic/supportive treatments.

Patients with a suspected tumor were evaluated by a multi-disciplinary team to determine whether surgery was recommended and its timing. Operations were performed by surgeons of West China Hospital of West China Second University Hospital. Patients were discharged following a surgery or transferred to a neurological intensive care unit for post-surgical immunotherapy depending on their neurological symptoms. Pathological diagnoses were made, following a surgery.

### Data Collection and Definition

Patients' clinical characteristics during hospitalization were extracted from medical records and included epidemiologic data (sex and age), clinical data (date of onset, date of admission, and typical symptoms), biological data (CSF antibody titers, CSF cell count, glucose/protein/IgG synthesis rates, and IgG index), auxiliary examination (MRI/CT results, ultrasound results, electroencephalography [EEG] results), and clinical management data (treatment administered, date of immunotherapy, and date of surgery). All data were collected by clinicians using a standardized form. Follow-up visits were conducted by a clinician every 3 months from clinical onset over the telephone. Patients' neurological and psychiatric sequelae were questioned, and patients who reported worsening or new onset of the eight major groups of symptoms were requested to attend the neurological clinic to evaluate the possibility of relapse and undergo further investigations if necessary.

Outcomes were assessed using the modified Rankin scale (mRS) (12). Evaluations were carried out face-to-face by neurologists during hospitalization and by patients' or guardians' responses over the telephone, following the discharge. Relapse was defined as a worsening or new onset of previous symptoms, occurring after at least 2 months of improvement or stabilization and was confirmed by antibodies detected in the CSF. Clinical improvement was defined as a decrease of 1 or more in mRS score (2). A long-term favorable outcome was defined as an mRS score ≤ 2 (2).

### Statistical Analysis

For all statistical analyses, SPSS 20 (SPSS Inc., Chicago, IL, USA) was used. Quantitative statistics are reported as means ± *SD*s (normally distributed) or medians (interquartile ranges [IQR]). Student's *t*-tests were performed for comparisons of continuous variables. Chi-squared tests or Fisher's exact tests were performed for comparisons of categorical variables. Wilcoxon's test was used to analyze rank variables. A two-tailed *p* < 0.05 was considered significant.

### Ethics

This study was approved by the West China Hospital of the Sichuan University Research Ethics Committee. Informed consent was obtained from all patients.

## Results

### Clinical Characteristics

A total of 192 patients were included in this study, among whom 107 (55.7%) were women. The mean age of patients was 29.44 ± 13.01 years (range 9–78 years, IQR 19–37 years). Furthermore, 21 (10.9%) patients had teratoma, among whom 19 (90.5%) had an ovarian teratoma and two (9.5%) had a mediastinal teratoma. The prevalence of teratoma in women with anti-NMDAR encephalitis was 18.7%. Pathologic subtypes included mature teratoma in 18 (85.7%) patients, immature teratoma in two (9.5%) patients, and mixed germ cell tumor in one (4.8%) patient. The demographic and clinical characteristics are shown in Table 1. Table 2 shows the clinical characteristic of the 21 patients with teratoma.

**Table 1:** Clinical features of patients with and without teratoma.

	**Total, n (%)**	**With teratoma, n (%)**	**Without teratoma, n (%)**	***P*-value**
Quantity	192	21	171	-
Age, years (mean ± SD)	29.44 ± 13.01	24.62 ± 7.61	30.03 ± 13.46	0.009^*^
Sex (female)	107 (55.7)	20 (95.2)	87 (50.9)	<0.001^#^
Psychiatric symptoms	175 (91.1)	19 (90.5)	156 (91.2)	0.829^#^
Seizure	153 (79.7)	18 (85.7)	135 (78.9)	0.660^#^
Speech impairment	47 (24.5)	8 (38.1)	39 (22.8)	0.124^#^
Dyskinesias/movement disorders	79 (41.1)	7 (33.3)	72 (42.1)	0.441^#^
Autonomic instability	92 (47.9)	13 (61.9)	79 (46.2)	0.174^#^
Memory deficits	157 (81.8)	15 (71.4)	142 (83.0)	0.193^#^
Decreased consciousness	77 (40.1)	15 (71.4)	62 (36.3)	0.002^#^
Cognitive disorder	126 (65.6)	13 (61.9)	113 (66.1)	0.650^#^
Central hypoventilation	40 (20.8)	11 (52.4)	29 (17.0)	<0.001^#^
Baseline mRS score	4 (4.5)	5 (4.5)	4 (4.5)	0.004^+^
Abnormal MRI findings	74/181 (40.9)	7/21 (33.3)	67/160 (41.9)	0.454^#^
Abnormal EEG findings	141/170 (82.9)	16/18 (88.9)	125/152 (88.2)	0.705^#^
Antibody titer in cerebrospinal fluid
1:1–1:10	44	1	43	0.021^+^
1:10–1:100	115	13	102	
1:100–1:1000	33	7	26	

* *Student's t-test*.

# *chi-squared or Fisher's exact tests*.

+ *Wilcoxon's test*.

**Table 2:** Clinical features of patients with teratoma.

**No**.	**Sex**	**Age**	**Prodrome**	**Initial symptoms**	**Baseline mRS score**	**Pathology**
Case 1	f	18	headache	seizures	5	mediastinal mature teratoma
Case 2	f	17	fever	seizures	5	ovarian mature teratoma
Case 3	m	25	headache/nausea	psychiatric symptoms	5	mediastinal mixed germ cell tumor (choriocarcinoma and teratoma)
Case 4	f	19	headache/fever	psychiatric symptoms	5	ovarian mature teratoma
Case 5	f	22	-	psychiatric symptoms	3	ovarian mature teratoma
Case 6	f	28	-	psychiatric symptoms	5	ovarian mature teratoma
Case 7	f	31	headache/fever	seizures	5	ovarian mature teratoma
Case 8	f	35	-	psychiatric symptoms	5	ovarian immature teratoma (WHO III)
Case 9	f	40	dizziness	psychiatric symptoms	4	ovarian mature teratoma
Case 10	f	18	headache/fever	seizures	5	ovarian mature teratoma
Case 11	f	20	headache	psychiatric symptoms	5	ovarian mature teratoma
Case 12	f	20	finger numbness	seizures	5	ovarian mature teratoma
Case 13	f	22	sleep disorder	psychiatric symptoms	4	ovarian mature teratoma
Case 14	f	43	-	seizures	4	ovarian immature teratoma (WHO III)
Case 15	f	17	sleep disorder	psychiatric symptoms	4	ovarian mature teratoma
Case 16	f	29	headache/fever	psychiatric symptoms	5	ovarian mature teratoma
Case 17	f	16	upper-respiratory-tract symptoms	psychiatric symptoms	4	ovarian mature teratoma
Case 18	f	26	-	psychiatric symptoms	3	ovarian mature teratoma
Case 19	f	26	headache/fever	psychiatric symptoms	5	ovarian mature teratoma
Case 20	f	19	-	seizures	5	ovarian mature teratoma
Case 21	f	26	-	psychiatric symptoms	5	ovarian mature teratoma

The mean age of the teratoma cohort was younger than that of patients without teratoma (24.62 vs. 30.03 years, *p* = 0.009). Psychiatric symptoms (91.1%), memory deficits (81.8%), and seizures (79.7%) were the most dominant symptoms. Central hypoventilation (52.4 vs. 17%, *p* < 0.001) and decreased consciousness (71.4 vs. 31.3%, *p* = 0.002) were significantly more frequent in patients with teratoma than in those without. Gynecological symptoms were rare, wherein only one patient complained of a prolonged intermenstrual period, which may be related to the teratoma. One patient had a medical history of teratoma removal, and relapsed teratoma was detected. Patients with teratoma tended to score lower on the mRS during the acute phase than did patients without teratoma (*p* = 0.004). Approximately one-third of patients showed abnormal MRI findings, and over 80% of patients showed abnormal EEG findings. CSF findings suggested higher antibody titer in patients with teratoma (*p* = 0.021).

### In-Hospital Management

First-line immunotherapies were administered to all patients with teratoma and 167 (97.7%) patients without teratoma. Patients with teratoma tended to use more turns of first-line immunotherapies (*p* = 0.013). The use of second-line immunotherapies did not significantly differ between the two groups. Among the 21 patients with teratoma, 17 (90%) patients underwent surgery during the acute phase before clinical improvement, and one patient underwent surgery before immunotherapy was administered. Table 3 shows the management of teratoma patients.

**Table 3:** Management and outcomes of patients with teratoma.

**No**.	**Intravenous immunoglobulin turns**	**Intravenous methylprednisolone turns**	**Second-line immunotherapy**	**Duration from onset to immuontherapy (days)**	**Duration from onset to removal surgery (days)**	**mRS score at surgery**	**mRS score at final follow-up**
Case 1	1	1	rituximab	15	165	2	0
Case 2	2	1	-	20	98	3	1
Case 3	2	-	-	7	87	5	6
Case 4	3	1	-	7	50	5	2
Case 5	1	-	-	21	272	0	0
Case 6	2	1	-	10	58	5	0
Case 7	3	3	-	15	221	0	0
Case 8	1	-	-	20	27	5	1
Case 9	1	3	-	15	39	4	0
Case 10	2	-	-	2	23	5	0
Case 11	3	1	-	14	42	5	2
Case 12	2	-	-	20	38	5	0
Case 13	1	1	-	21	34	4	0
Case 14	1	-	-	22	15	4	2
Case 15	2	1	-	10	19	4	0
Case 16	4	1	rituximab	20	125	5	1
Case 17	1	1	cyclophosphamide	5	32	4	1
Case 18	1	1	-	10	27	3	0
Case 19	2	2	rituximab	35	58	5	6
Case 20	2	-	-	20	47	5	1
Case 21	1	2	-	7	22	5	1

### Follow-Up and Outcome

The median follow-up period was 46 months (6–91 months). During the follow-up period, two (9.5%) patients with teratoma and 11 (6.4%) patients without teratoma died. Among the two patients with teratoma who died, one died because of pulmonary metastasis of the tumor (mixed germ cell tumor) and secondary respiratory failure, and the other died because of pancreatitis, pulmonary infection, septic shock, and multiple organ dysfunction syndromes. In total, 19 (90.5%) teratoma patients and 151 (88.3%) patients without teratoma achieved a favorable clinical outcome at the final follow-up. The clinical management and outcomes of patients are shown in Table 4.

**Table 4:** Management and outcomes of patients with and without teratoma.

	**Total**,	**With teratoma**,	**Without teratoma**,	***P*-values**
	**n (%)**	**n (%)**	**n (%)**	
Intravenous immunoglobulin	172 (89.6)	21 (100)	151 (88.3)	0.136^#^
Intravenous methylprednisolone	122 (63.5)	14 (66.7)	108 (63.2)	0.940^#^
Second-line immunotherapy	16 (8.3)	4 (19)	12 (7)	0.143^#^
Intravenous first-line immunotherapy turns (median, IQR)	2 (1–5)	2 (1–4)	2 (1–5)	0.013^+^
mRS score at final follow up	0 (0–1)	1 (0–1)	0 (0–1)	0.864^+^
Death	13 (6.8)	2 (9.5)	11 (6.4)	0.943^#^
Relapse	38 (19.8)	1 (4.8)	37 (21.6)	0.123^#^

# *chi-squared or Fisher's exact tests*.

+ *Wilcoxon's test*.

**Figure 1:**
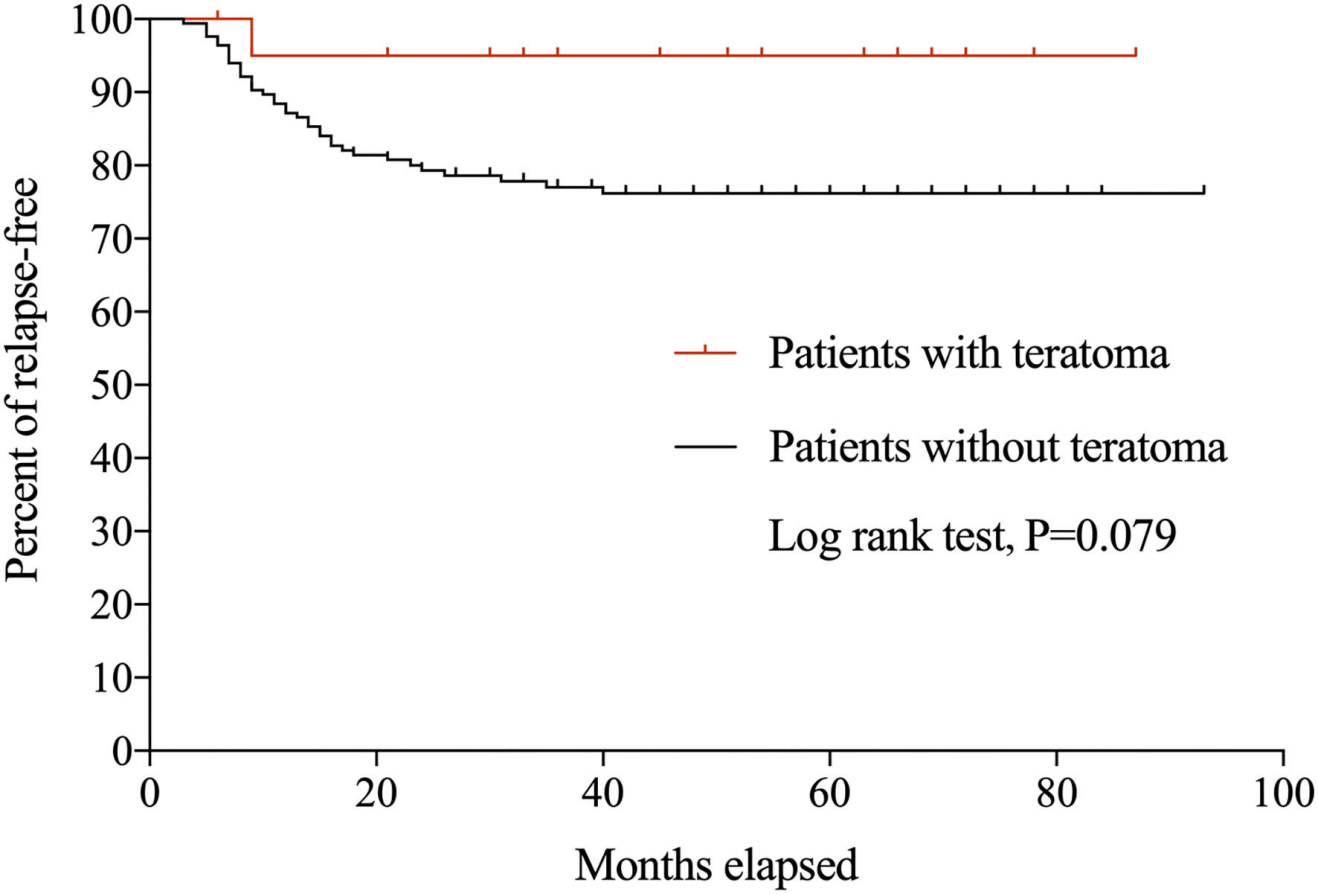
Kaplan-Meier curves for anti-NMDAR encephalitis patients with and without teratoma.

During the follow-up period, 39 patients experienced 47 relapses. Only one (4.8%) patient with teratoma relapsed at 7 months after initial onset, with recurrent seizures and memory deficits and CSF IgG titer of 1:100. Symptoms were controlled swiftly following the administration of IVIg, and there was no evidence of relapsed teratoma. Figure 1 shows the Kaplan-Meier curves of anti-NMDAR encephalitis patients with and without teratoma.

## Discussion

We found that most patients with teratoma recovered slowly. However, favorable clinical outcomes were achieved over long-term follow-up, although mild sequelae may last several years. Immunotherapy was comparably ineffective in patients with teratoma before surgery, but effectiveness improved following removal surgery. Patients with teratoma presented with a more acute onset, more severe neurological symptoms, and higher IgG titer, than those without teratoma. Therefore, earlier and more immunotherapy turns were recommended for these patients.

The prevalence of teratoma in this study was comparably lower than that in previous studies. Titulaer et al. reported a prevalence of 211/577 patients, who were predominantly Asian and African-American (2). On the other hand, Xu et al. reported a prevalence of 42/143 women patients in a Chinese cohort (13). In our study, the prevalence of teratoma was 21/192 patients and was higher in younger individuals. This is consistent with the findings of Titulaer et al. (2) in which teratoma primarily affects individuals aged between 12 and 45 years, recommending more comprehensive inspections for teratoma in female youth. Anti-NMDAR encephalitis triggered by extra-ovarian teratoma, especially mediastinum teratoma, was detected in 2/21 patients with teratoma in our cohort. Overlooked extra-ovarian teratoma may result in delayed diagnosis in some cases (14). In addition, the teratoma cohort presented with more severe neurological sequelae, with greater disturbance of consciousness and central hypoventilation, and higher anti-NMDAR antibody titer in the CSF, which is similar to the report by Gresa-Arribas et al. (15). Accordingly, wider use of ventilators and intensive care has also been reported in this patients group (16). MRI and EEG during the acute phase showed non-specific changes, with limited significance for diagnoses. However, the evaluation using positron emission tomography was recommended in several cases.

Immunotherapy is a crucial element of autoimmune encephalitis treatment. The combination of steroids, intravenous immunoglobulins, and plasma exchange is recommended, and second-line therapy should be administered as soon as possible if first-line therapy is unsuccessful (17). In our study, patients with teratoma responded more poorly to immunotherapy and required more turns of immunotherapy than patients without teratoma. Persistent germinal center response in teratoma can produce NR1-IgG continuously (18). Single immunotherapy has a limited effect in patients with teratoma. However, a high proportion of patients with inadequate response to immunotherapy has been found to improve, following a surgery as reflected in the control of seizures and increased consciousness level. Dalmau et al. observed 105 anti-NMDAR encephalitis patients and reported an 80% response rate to first-line immunotherapy plus surgery in patients with teratoma, whereas the response rate in patients without teratoma was 48% (19). Improvement can be dramatic in some patients, and can even occur within a few days of surgery (20).

Surgery during the acute phase is strongly recommended for a good long-term prognosis. Lee et al. suggest that delayed surgery is associated with poor improvement over time (21). Moreover, Dalmau et al. reported that patients who do not undergo surgery have a higher mortality rate (20). Furthermore, a previous study found that patients who undergo tumor removal within 4 months have milder neurological deficits than those who undergo a delayed surgery (22). Although the safety of undergoing surgery during the acute phase is a significant concern, consistent with a previous study (16), there were no surgical complications during the perioperative period in our cohort. Two of our patients died because of multiple organ failure due to anti-NMDAR encephalitis; however, there was no evidence to indicate that surgery hastened death in these patients.

The detection of teratoma during the early phase is important for medical management. CT and MRI have higher sensitivity than ultrasound for teratoma screening and thus are recommended for patients with anti-NMDAR encephalitis (23). However, patients with anti-NMDAR encephalitis have smaller teratoma with fewer teeth, less calcification, and a smaller fat-occupied space, which makes the detection of teratoma challenging (24). Indeed, Lee et al. reported that diagnosis of teratoma was missed in 26.1% of patients during initial pelvis CT, even when combined with MRI (21). Thus, continual reassessment for teratoma in patients who show no significant improvement with immunotherapy or those who relapse repeatedly is recommended. Although delayed surgery is criticized by many (21), our study indicated that patients can benefit from surgery, even with a delay of over 6 months.

Patients with anti-NMDAR encephalitis along with teratoma have benign prognoses, with a low relapse rate and mild sequelae. The teratoma group had long-term prognoses similar to patients without teratoma in terms of relapse, mortality, and mRS score, regardless of more dreadful onset. However, a lower relapse rate in patients with teratoma has been reported previously (16). One study reported that relapsed or residual teratoma can induce relapse (25), although we found no evidence of relapsed teratoma in patients who relapsed in this study.

Our study has several limitations. Firstly, our sample size of the single-center study was small. A multicenter study would increase the sample size and reduce selection bias. Secondly, the evaluation of prognoses during the post-surgical follow-up was based primarily on patients' subjective descriptions, and regular anti-NMDAR antibody tests and cranial MRI were not performed for further analysis.

In conclusion, removal surgery to treat anti-NMDAR encephalitis patients with teratoma is effective. Although anti-NMDAR encephalitis patients with teratoma had more serious medical conditions than patients without teratoma, timely removal surgery enabled favorable long-term outcomes. Comprehensive assessments are required for early tumor detection and timely management, especially in patients who respond poorly to immunotherapy.

## Data Availability Statement

The raw data supporting the conclusions of this article will be made available by the authors, without undue reservation.

## Ethics Statement

The studies involving human participants were reviewed and approved by West China Hospital of Sichuan University Research Ethics Committee. Written informed consent to participate in this study was provided by the participants' legal guardian/next of kin.

## Author Contributions

HZ: drafting of the manuscript for content, analysis and interpretation of data. WX and WL: revision of the manuscript for content. XL: major role in the acquisition of data. DZ and XW: study concept, design, and revision of the manuscript for content. All authors contributed to the article and approved the submitted version.

## Funding

This work was supported by grants from the National Natural Science Foundation of China (Grant Nos. 81871017 and 81901320), the Chengdu Science and Technology Bureau (2021-YF05-00846-SN), and Chengdu Science and Technology Bureau (2019-YF09-00215-SN).

## Conflict of Interest

The authors declare that the research was conducted in the absence of any commercial or financial relationships that could be construed as a potential conflict of interest.

## Publisher's Note

All claims expressed in this article are solely those of the authors and do not necessarily represent those of their affiliated organizations, or those of the publisher, the editors and the reviewers. Any product that may be evaluated in this article, or claim that may be made by its manufacturer, is not guaranteed or endorsed by the publisher.
